# GAF domain is essential for nitrate-dependent AtNLP7 function

**DOI:** 10.1186/s12870-022-03755-x

**Published:** 2022-07-25

**Authors:** Jie Wu, Ying Song, Zi-Sheng Zhang, Jing-Xian Wang, Xuan Zhang, Jian-Ye Zang, Ming-Yi Bai, Lin-Hui Yu, Cheng-Bin Xiang

**Affiliations:** 1 Division of Life Sciences and Medicine, Division of Molecular & Cell Biophysics, Hefei National Science Center for Physical Sciences at the Microscale, MOE Key Laboratory for Membraneless Organelles and Cellular Dynamics, University of Science and Technology of China, The Innovation Academy of Seed Design, Chinese Academy of Sciences, Hefei, 230027 Anhui Province China; 2 The Key Laboratory of Plant Development and Environmental Adaptation Biology, Ministry of Education, School of Life Sciences, Shandong University, Qingdao, 266237 Shandong Province China; 3 State Key Laboratory of Crop Stress Biology for Arid Areas and Institute of Future Agriculture, Northwest A&F University, Yangling, 712100 Shanxi China

**Keywords:** AtNLP7, Nitrate deficiency signaling, Nuclear localization, GAF domain, ROS

## Abstract

Nitrate is an essential nutrient and an important signaling molecule in plants. However, the molecular mechanisms by which plants perceive nitrate deficiency signaling are still not well understood. Here we report that AtNLP7 protein transport from the nucleus to the cytoplasm in response to nitrate deficiency is dependent on the N-terminal GAF domain. With the deletion of the GAF domain, AtNLP7^ΔGAF^ always remains in the nucleus regardless of nitrate availability. AtNLP7 ^ΔGAF^ also shows reduced activation of nitrate-induced genes due to its impaired binding to the nitrate-responsive *cis*-element (NRE) as well as decreased growth like *nlp7-1* mutant. In addition, AtNLP7^ΔGAF^ is unable to mediate the reduction of reactive oxygen species (ROS) accumulation upon nitrate treatment. Our investigation shows that the GAF domain of AtNLP7 plays a critical role in the sensing of nitrate deficiency signal and in the nitrate-triggered ROS signaling process.

**Supplementary Information:**

The online version contains supplementary material available at 10.1186/s12870-022-03755-x.

## Introduction

Nitrate is not only an essential mineral element for plant growth, but also a signal molecule involved in many important developmental processes. Plants have evolved sophisticated mechanisms to respond to nitrate triggering a cascade of consequent reactions [1–4]. Recent studies have shown that *NIN-like proteins* (*NLPs*) function as key transcription factors of primary nitrate responses [5–11]. Nitrate provision signaling is known to promote nuclear localization of *Arabidopsis* AtNLP7 through Ca^2+^ sensor CPKs [12]. Nevertheless, how nitrate deficiency signaling is relayed to *AtNLP7* remains unclear.

Members of the NLP family all contain an amino-terminal GAF domain, an intermediate RWP-RK domain, and a carboxy-terminal Phox and Bem1 (PB1) domain [13]. Additionally, a nuclear export signal domain is predicted in the N-terminus [6, 14]. The RWP-RK domain is characterized by the conserved five-amino acid sequence Arg-Trp-Pro-X-Arg-Lys, which binds nitrate-responsive cis-elements (NREs) [15, 16]. The PB1 domain is mainly involved in the interaction between proteins [17], such as the interactions of NLP-NLP and NLP-TEOSINTE BRANCHED1/CYCLOIDEA/PROLIFERATING CELL FACTOR1-20 (TCP20) [18]. Furthermore, the PB1 domain of NLP transcription factors may mediate homo- and hetero-oligomerization, thereby regulating the expression of target genes in the presence of nitrate [19].

The GAF domain is widely present in different types of proteins and was named after cGMP-regulated phosphodiesterase, certain adenylyl cyclases and FhlA were found to contain this domain [20]. Most GAF domains can bind multiple small molecular ligands and participate in various signal transduction pathways throughout the life cycle [20–23], such as the GAF domain of NreA in *Staphylococcus* can form hydrophobic pocket that directly bind nitrate [20–23]. In higher plants, the proteins containing GAF domains are usually involved in light absorption and ethylene signal transduction [24, 25]. Whereas, the role of the GAF domain in NLPs remains unclear.

It is known that AtNLP7 responds to nitrate through a nuclear retention mechanism [6]. AtNLP7 is present in both the nucleus and cytoplasm under normal conditions. When nitrate is deficient, AtNLP7 locates in the cytoplasm, then relocates to the nucleus after a few minutes of re-supply of nitrate [6]. Studies have shown that the N-terminal region of AtNLP6 is responsible for receiving nitrate signals [7]. In addition, the phosphorylation of Ser205 at the N-terminus of AtNLP7 is necessary for AtNLP7 to translocate to the nucleus in response to nitrate signals [12]. It is known that AtNLP7 senses the nitrate signal, then moves into the nucleus and play its regulatory role. However, the underlying mechanism by which AtNLP7 senses the nitrate deficiency signal and then moves out of the nucleus remains unknown.

In this study, we revealed that the GAF domain is critical for the transport of AtNLP7 from the nucleus to the cytoplasm. With the loss of the GAF domain, AtNLP7^ΔGAF^ remained in the nucleus, while the protein lost its ability to bind to NRE or to increase plant nitrogen (N) use efficiency. In addition, AtNLP7^ΔGAF^ lost its ability to mediate the reduction of ROS accumulation upon nitrate treatment. Our investigation shows that the N-terminal GAF domain is required for AtNLP7 protein translocation from the nucleus to the cytoplasm in response to nitrate deficiency.

## Results

### GAF domain is required for AtNLP7 nuclear exportation in response to nitrate deficiency

**Fig. 1:**
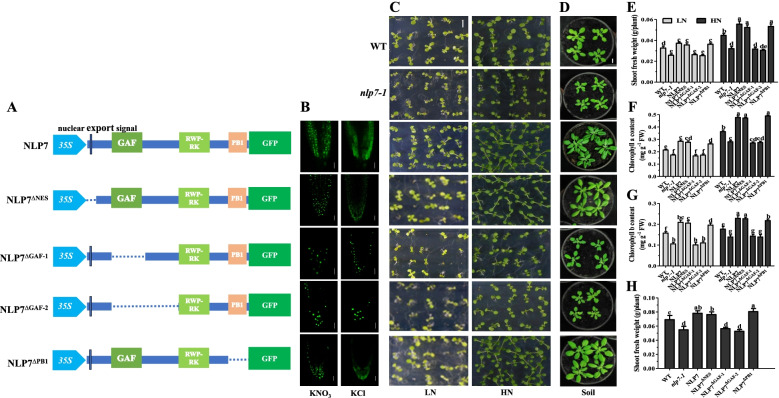
The GAF domain is required for the transport of AtNLP7 from the nucleus to the cytoplasm in response to nitrate deficiency and affects plant growth. **A** Domain structures schematic of the GFP fusions for the AtNLP7 derivatives. NLP7: construct used to express the full-length AtNLP7 protein driven by CaMV *35S* promoter. NLP7^∆NES^: construct used to express the AtNLP7 protein with deleted nuclear export sequence driven by CaMV *35S* promoter. NLP7^∆GAF−1^: construct used to express the AtNLP7 protein with deleted GAF domain driven by CaMV *35S* promoter. NLP7^∆GAF−2^: construct used to express the AtNLP7 protein with deleted region from GAF domain to RWP-RK domain driven by CaMV *35S* promoter. NLP7.^∆PB1^: construct used to express the AtNLP7 protein with deleted PB1 domain driven by CaMV *35S* promoter. Each construct contained the GFP in the C-terminal. **B** Nucleocytosolic shuttling of AtNLP7 derivatives. Confocal imaging was performed on *nlp7-1* seedlings expressing different fusion proteins. Seedlings growing on medium with 10 mM KNO_3_ for five days were transferred to N-free medium for two days then treated with 10 mM KNO_3_ or KCl for one hour. Scale bars, 20 μm. **C** Plants grown with a density of 20 plants per line on 1 mM (LN) or 10 mM (HN) KNO_3_ modified MS medium for 10 days. Scale bars, 0.5 cm. **D** Image of 3-week-old AtNLP7 derivate lines, *nlp7-1* and WT plants grew in soil. Scale bars, 1 cm. **E**–**G** The shoot fresh weight (**E**), chlorophyll a (**F**) and b (**G**) contents of the 10-day-old plants grown as in C. Values are the mean ± SD of three independent replications each containing 20 plants per genotype. *P* values are from the one-way ANOVA (The letters indicate significant differences. *P* < 0.05). **H** Shoot fresh weight of the plants grown as in **D**. Values are the mean ± SD of four independent replications each containing 6 plants per genotype (The letters indicate significant differences. *P* < 0.05)

To investigate which domain is responsible for the nitrate deficiency-triggered AtNLP7 relocation from the nucleus to cytoplasm, we generated transgenic lines expressing a series of truncated AtNLP7 domains fused with the green fluorescence protein (GFP) in the *nlp7-1* background. The nuclear export signal (NES), GAF domain and PB1 domain of AtNLP7 were deleted respectively as shown in Fig. 1A. The expression of *AtNLP7* was significantly increased in all complementary lines compared to *nlp7-1* and wild-type (WT) plants (Fig. S1).

In the presence of nitrate, AtNLP7-GFP was mainly located in the nucleus. Removing nitrate within hours led to the relocation of AtNLP7-GFP into the cytoplasm (Fig. 1B). Interestingly, we found that when the NES of AtNLP7 was deleted (NLP7^ΔNES^), the protein could still be transported between the nucleus and cytoplasm, indicating that the NES pathway is not the only way for AtNLP7 to be transported out of the nucleus. Similarly, when the PB1 domain was deleted (NLP7^ΔPB1^), the protein could also be transported between the nucleus and cytoplasm (Fig. 1B).

Surprisingly, two AtNLP7 deletion variants that did not contain the GAF domain (NLP7^ΔGAF−1^ and NLP7^ΔGAF−2^) remained in the nucleus regardless of nitrate presence in the surrounding environment, suggesting that these variants could be imported into the nucleus but could not be exported out of it (Fig. 1B). Taken together, these results indicate that the GAF domain, but not the NES and the PB1 domain, is responsible for sensing the nitrate deficiency signal that mediates AtNLP7 nuclear exportation.

### NLP7^ΔGAF^ variants fail to complement ***nlp7-1*** mutant

In order to explore the function of different domains of AtNLP7, the generated lines were grown on MS medium with nitrate as the only N source. The *nlp7-1* mutants exhibited N-deficient phenotype regardless nitrate level, and the phenotype has been restored by expressing *35S*:AtNLP7-GFP construct (NLP7) (Fig. 1C). Compared with *nlp7-1* plants, overexpression of NLP7^ΔNES^ could increase shoot biomass regardless nitrate level, as well as the overexpression of NLP7^ΔPB1^ (Fig. 1E). However, the transgenic lines overexpressing NLP7^ΔGAF−1^ and NLP7^ΔGAF−2^ exhibited impaired growth even under nitrate-rich conditions (Fig. 1C, E). When grown in soil, NLP7^ΔGAF−1^ and NLP7^ΔGAF−2^ plants showed N-stressed phenotypes with lower biomass whereas NLP7^ΔNES^ and NLP7^ΔPB1^ plants exhibited a normal phenotype (Fig. 1D, H). We also found that chlorophyll contents increased significantly in NLP7^ΔNES^ and NLP7^ΔPB1^ plants while reduced dramatically in NLP7^ΔGAF−1^ and NLP7^ΔGAF−2^ plants compared to the WT under both low and high nitrate conditions (Fig. 1F, G). All these results indicate that the mutated forms of AtNLP7 without GAF domain lost its ability to restore the phenotype of the *nlp7-1* plant.

### NLP7^ΔGAF^ plants show downregulated expression of nitrate-responsive genes and impaired nitrate assimilation

**Fig. 2:**
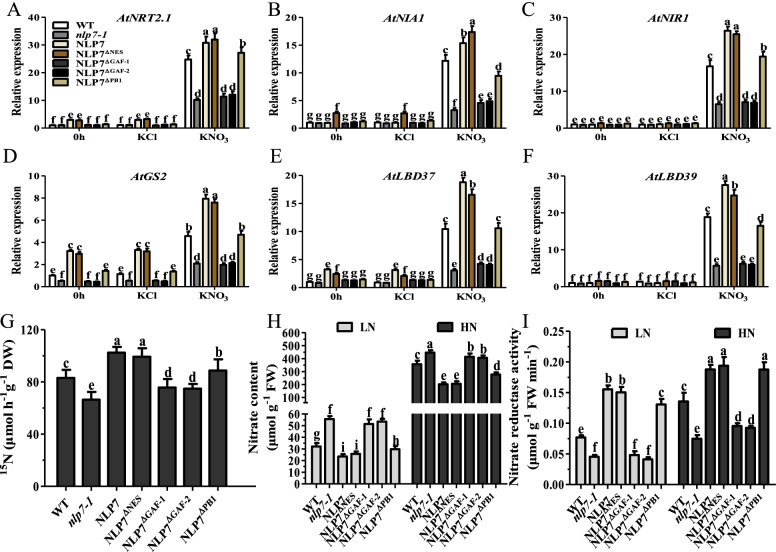
Effects of mutations in different domains of AtNLP7 on nitrate-inducible gene expression and nitrate assimilation ability. **A-F** Seedlings of WT, *nlp7-1* and the complementation lines were grown on N-free medium for 7 days and then treated with 10 mM KNO_3_ for 1 h. Transcript levels were normalized against *AtUBQ5* expression. RT-qPCR data are mean ± SD (*n* = 3). *P* values are from the one-way ANOVA (The letters indicate significant differences. *P* < 0.05). *NRT2.1*, nitrate transporter 2.1; *NIA1*, nitrate reductase 1; *NIR1*, nitrite reductase 1; *GS2*, glutamine synthetase 2; *LBD*, lateral organ boundary domain. **G** ^15^NO_3_^−^ uptake activity assay. 10-day-old seedlings were labeled with 5 mM ^15^NO_3_^−^ for 1 h and the amount of ^15^NO_3_^−^ taken into the plants was measured. Values are the mean ± SD of three replications each containing 30 plants per genotype (The letters indicate significant differences. *P* < 0.05). DW, dry weight. **H**, **I** Content of nitrate (**H**) and enzyme activity of nitrate reductase (**I**) in the plants grown under different nitrate conditions. 14-day-old seedlings grown on agar medium with 1 mM (LN) or 10 mM (HN) KNO_3_ were used for metabolite analyses as described in Material and Methods. Values are the mean ± SD of three replications each containing 15 plants per genotype (The letters significant differences. *P* < 0.05). FW, fresh weight

To assess the ability of truncated AtNLP7 to affect nitrate-responsive gene expression, we examined the transcriptional levels of these genes without nitrate and with re-supply of nitrate for one hour. The expression of typical nitrate-responsive genes, containing nitrate transporter gene *NITRATE TRANSPORTER 2.1* (*NRT2.1*), nitrate assimilation genes *NITRATE REDUCTASE 1* (*NIA1*) and *NITRITE REDUCTASE 1* (*NIR1*), glutamine synthetase gene *GLUTAMINE SYNTHETASE 2* (*GS2*) and two transcription factor genes *LOB DOMAIN-CONTAINING PROTEIN 37* (*LBD37)* and *LBD39*, was analyzed. Induction of these genes in *nlp7-1* is approximately half the levels seen in WT seedlings (Fig. 2A-F). However, expressing wild-type *AtNLP7* with the CaMV-*35S* promoter in *nlp7-1* (NLP7 plants) strongly restored the expressive levels of these genes in response to nitrate. Nitrate-induced expression of all genes analyzed also showed a similar increase in NLP7^ΔNES^ plants (Fig. 2A-F). Nevertheless, transcription levels of these genes were basically the same in *nlp7-1* and NLP7^ΔGAF^ seedlings after nitrate addition (Fig. 2A-F). In addition, the expression of these genes in the NLP7^ΔPB1^ plants were similar to those in the WT plants under either N-free or nitrate-supplied conditions (Fig. 2A-F). These results suggest that the GAF domain plays an essential role for AtNLP7 regulation of nitrate-inducible genes.

We further investigated N absorption and assimilation by analyzing ^15^ N-nitrate absorption and found that ^15^ N accumulation in NLP7, NLP7^ΔNES^ and NLP7^ΔPB1^ plants was observably more than that in WT while much less in NLP7^ΔGAF−1^ and NLP7^ΔGAF−2^ plants (Fig. 2G). We next analyzed the nitrate content of different lines under low and high nitrate conditions. As expected, the *nlp7-1* mutant accumulated more nitrate due to the weak nitrate reductase (NR) activity [5]. The nitrate content in NLP7^ΔGAF−1^ and NLP7^ΔGAF−2^ plants also increased significantly while decreased dramatically in NLP7^ΔNES^ and NLP7^ΔPB1^ plants (Fig. 2H). We then compared the NR activity of the transgenic lines with WT and discovered that NR activities were lower in NLP7^ΔGAF−1^ and NLP7^ΔGAF−2^ plants while higher in NLP7, NLP7^ΔNES^ and NLP7^ΔPB1^ plants under different nitrate conditions (Fig. 2I). Overall, our analysis suggests that the GAF domain is required for the function of AtNLP7.

### NLP7^ΔGAF^ fails to bind its targets in vivo and activate their expression

**Fig. 3:**
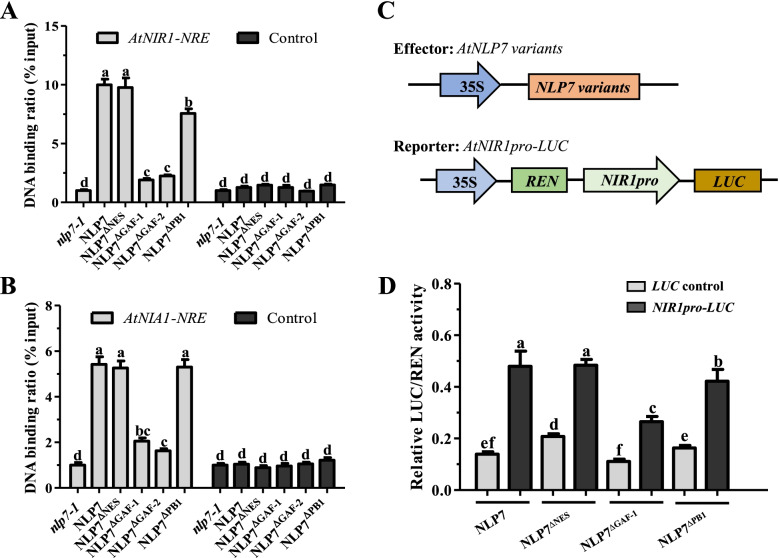
AtNLP7^ΔGAF^ loses its ability to bind NRE and fails to activate the expression of its target genes. **A**, **B** ChIP–qPCR enrichment of NRE-containing promoter fragments (relative to input) from *AtNIR1* (**A**) and *AtNIA1* (**B**) in different AtNLP7 domain deletion mutants. Data are mean ± SD (*n* = 3). The letters indicate significant differences (*P* < 0.05). **C** Schematic representation of the effector and reporter constructs used in the transactivation assay. **D** Transient transactivation assays. Different AtNLP7 domain deletion variants were used to activate *AtNIR1* promoter-LUC fusion construct as described in the Materials and Methods. Renilla luciferase gene (*REN*) used as an internal control. Data are mean ± SD (*n* = 3). The letters indicate significant differences (*P* < 0.05)

To reveal the function of the GAF domain in AtNLP7 target gene activation, we conducted chromatin immunoprecipitation (ChIP) quantitative PCR and identified the association of AtNLP7 with NREs from the *AtNIR1* and *AtNIA1* promoters in vivo. Compared with NLP7, the binding ability of NLP7^ΔNES^ to NRE element was not significantly changed, but NLP7^ΔPB1^ was slightly impaired. In contrast, NLP7^ΔGAF−1^ and NLP7^ΔGAF−2^ almost lost the function to combine NRE element (Fig. 3A, B). This result was further verified with transient transactivation assay, in which AtNLP7^ΔGAF^ weakened the activation of the *AtNIR1* promoter (Fig. 3C, D).

### The GAF domain of AtNLP7 is required for the nitrate-induced reduction of ROS accumulation

**Fig. 4:**
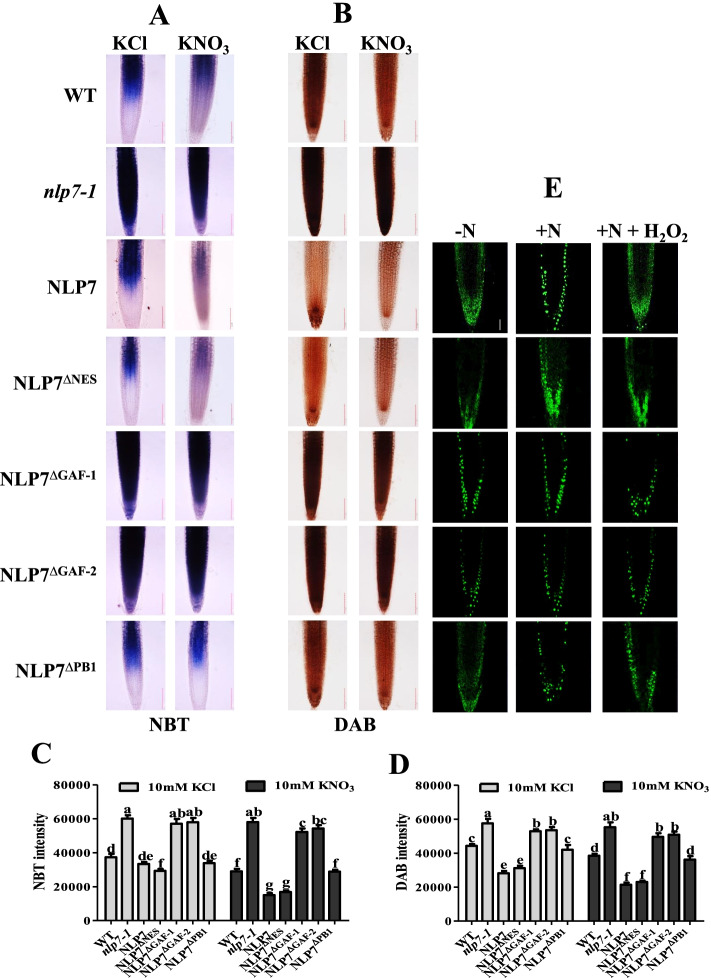
Nitrate inhibits the accumulation of ROS through the GAF domain of AtNLP7. **A**, **B** NBT and DAB staining showed that nitrate treatment significantly reduced the contents of ROS in the primary root of *nlp7-1* and *AtNLP7*^*ΔGAF*^ plants. Bar = 100 μm. Plants growing on medium with 10 mM KNO_3_ for five days were transferred to N-free medium for two days then treated with 10 mM KCl or KNO_3_ for one hour. **C**, **D** Quantification of NBT and DAB intensities by Image J. Data are mean ± SD (*n* = 10). The letters indicate significant differences (*P* < 0.05). **E** ROS treatment attenuated nitrate-induced nuclear localization of AtNLP7 but not AtNLP7^ΔGAF^. Plants growing on medium with 10 mM KNO_3_ for five days were transferred to N-free medium for two days then treated with 10 mM KNO_3_ or 10 mM KNO_3_ + 0.5 mM H_2_O_2_ for one hour. Bar = 50 μm

ROS has recently been found to play an important role in nitrate signal transduction in *Arabidopsis thaliana* [26–32]. In order to confirm whether nitrate modulates ROS contents through *AtNLP7*, we used nitroblue tetrazolium (NBT) and 3,3'-diaminobenzidine tetrahydrochloride (DAB) staining to detect the level of ROS in WT and different AtNLP7 truncated mutants with or without nitrate application. The results showed that nitrate treatment significantly reduced ROS levels in the primary root of NLP, NLP7^ΔNES^ and NLP7^ΔPB1^ plants compared with the WT, but there was no obvious change in the primary root of NLP7^ΔGAF−1^, NLP7^ΔGAF−2^ and *nlp7-1* plants (Fig. 4A-D), suggesting that nitrate-triggered ROS reduction is dependent on GAF domain.

To confirm the above results, we analyzed truncated AtNLP7-GFP proteins in the primary root of transgenic plants to determine whether H_2_O_2_ addition influenced the localization of AtNLP7. Nitrate treatment induced the nuclear localization of AtNLP7-GFP. Whereas, addition of H_2_O_2_ dramatically inhibited nitrate-induced translocation of NLP7-GFP and various truncated AtNLP7-GFP from the cytoplasm to the nucleus except NLP7^ΔGAF^ variants (Fig. 4E). These results indicate that the GAF domain is required for the H_2_O_2_ treatment-reduced movement of AtNLP7 from cytoplasm into nucleus.

## Discussion

*AtNLP7* has been shown to play a crucial role in the primary nitrate response [6, 9]. The subcellular localization of AtNLP7 is exclusively and quickly induced by nitrate [6]. Phosphorylation of AtNLP7 at Ser205 by Ca_2_^+^ receptor kinase AtCPK10/30/32 is required for nitrate-induced migration of AtNLP7 from the cytoplasm to the nucleus [12]. Whereas, the underlying mechanism by which AtNLP7 senses the nitrate deficiency signal and thus relocate from the nucleus to cytoplasm remains unclear. In this study, we demonstrated that AtNLP7^ΔGAF^ remained in the nucleus under nitrate-starvation conditions and lost its function as a transcriptional activator. Therefore, the GAF domain is responsible for sensing nitrate deficiency signal that mediates AtNLP7 nuclear exportation.

Most of the nucleocytoplasmic transport is a signal dependent process, requiring a sequence motif in the transported protein [14]. The presence of a predicted leucine-rich NES within AtNLP7 led us to examine whether this NES was responsible for the cytoplasmic localization of AtNLP7 during nitrate starvation. Leptomycin B (an inhibitor of Exportin1) treatment have been reported to inhibit the transport of NLP7–GFP from the nucleus to the cytoplasm when the seedlings are transferred from the nitrate-rich condition to the N-starvation [6]. Unexpectedly, we found that NLP7^ΔNES^ could still be transported between the nucleus and cytoplasm (Fig. 1B), indicating that the NES is dispensable for AtNLP7 to be transported out of the nucleus in response to nitrate deficiency signal.

The RWP-RK domain is required for AtNLP7 binding to NRE *cis*-elements of target genes but not for nitrate signaling [7, 15, 33, 34]. In the present study, reduced enrichment of NRE *cis*-elements in chromosome immunoprecipitation assays and attenuated levels of transcription in protoplast transient tests (Fig. 3), indicating that the GAF domain is also crucial for AtNLP7 binding to NRE. NLP7^Δ GAF^ significantly weakens its binding to the promoter of target genes and thereby its transcription-activating activity compared with wild-type NLP7. Because the GAF domain can bind low-molecular-weight ligands such as nitrate or form homodimer [20–23], it is possible that the specific spatial conformation of AtNLP7 resulting from such binding is necessary for its transcriptional activation activity.

The PB1 domain plays an important role in protein–protein interaction [17, 35]. Regulation of nitrate-dependent target gene expression by NLP transcription factors requires protein–protein interactions caused by the PB1 domain in plants [19]. Indeed, we showed that the deletion of the PB1 domain (NLP7^ΔPB1^) had a significant effect on the expression level of nitrate-induced genes compared with wild-type AtNLP7 (NLP7) (Fig. 2A-F), consistent with its growth phenotype (Fig. 1C-H) and other results (Figs. 3 and 4), although there was no impairment of nuclear export capacity in response to nitrate starvation (Fig. 1B).

H_2_O_2_ is produced during N deficiency and act as a potential messenger in the nitrogen starvation response [26, 27, 30, 31]. Recently, it has been reported that nitrate application decreases the accumulation of H_2_O_2_, while H_2_O_2_ suppresses nitrate signaling through modulating the nucleocytoplasmic shuttling of AtNLP7 [28]. We found that the GAF domain plays an important role in this process and reached similar conclusions (Fig. 4). The results of NBT and DAB staining showed that the addition of nitrate significantly reduced the content of ROS in WT, NLP7, NLP7^∆NES^ and NLP7^∆PB1^ seedlings compared with the nitrate starvation treatment. However, due to the deletion of GAF domain in AtNLP7, ROS accumulated in the absence of nitrate and could not be effectively reduced after nitrate addition, consistent with those in *nlp7-1* mutant (Fig. 4A-D). These results indicate that the GAF domain of AtNLP7 is required for nitrate-induced reduction of ROS. In addition, we also found that H_2_O_2_ addition decreases the nuclear localization of AtNLP7 triggered by nitrate in a GAF domain-dependent manner (Fig. 4E). H_2_O_2_ usually modulates the activity of the target proteins by oxidative modification of cysteine [36]. AtNLP7 GAF domain contains 2 cysteine residues (Cys273 and Cys296) that can be oxidized by H_2_O_2_, thereby sensing H_2_O_2_ signal to regulate AtNLP7 subcellular localization and subsequent events.

## Methods

### Plant materials and growth conditions

*Arabidopsis thaliana* ecotype Col-0 and *nlp7-1* (SALK_26134C) seeds were obtained from *Arabidopsis* Biological Resource Center with permission and used for the genetic transformation of the *AtNLP7* gene. 35S:NLP7^∆NES^-GFP, 35S:NLP7^∆GAF−1^-GFP, 35S:NLP7^∆GAF−2^-GFP and 35S:NLP7^∆PB1^-GFP constructs were made by inserting the different coding regions of AtNLP7 into pGWB5 through GATEWAY cloning system. In the construction of 35S:NLP7–GFP, the fragment containing the complete coding sequence of *AtNLP7* was cloned into pGWB5 to fuse with GFP. All transgenic lines were generated and screened as previously described [5].

The sterilization of the seeds and the growth conditions of the seedlings were as previously described [5–8, 11]. Seeds were sterilized with 15% bleach for 12 min, and then washed five times with sterile water. Sterilized seeds stratified at 4 °C for 2 days, and plated on solid medium containing 1% (w/v) sucrose and 0.6% (w/v) agar. Growth medium was modified on MS medium with KNO_3_ as sole N source: 1 mM (LN) nitrate medium (similar to MS except 20 mM KNO_3_ and 20 mM NH_4_NO_3_ was replaced with 19 mM KCl and 1 mM KNO_3_), 10 mM (HN) nitrate medium (similar to MS except 20 mM KNO_3_ and 20 mM NH_4_NO_3_ was replaced with 10 mM KCl and 10 mM KNO_3_), N-free medium (similar to MS except 20 mM KNO_3_ and 20 mM NH_4_NO_3_ was replaced with 20 mM KCl).

The seedlings for phenotypic analysis in Fig. 1 were grown on 1 mM (LN) and 10 mM (HN) medium for 10 days or in soil for 3 weeks, respectively. To analyze expression of nitrate-induced genes, seedlings were grown on N-free medium for 7 days and then treated with 10 mM KCl or KNO_3_ for 1 h.

To investigate different growth rates under different N conditions, seeds were germinated and grown on medium containing different concentrations of nitrate at 22 °C under 16-h light/8-h dark photoperiod. For evaluation the phenotype of soil-grown plants, seeds were germinated and grew in soil at 22 °C under 16-h light/8-h dark photoperiod.

### Subcellular localization assay

To investigate the nuclear-cytoplasmic shuttling of AtNLP7 with different domains, seedlings grown for 5 days on 10 mM KNO_3_ medium were transferred to N-free medium for 2 days and then treated with 10 mM KNO_3_ or KCl for 1 h. To analyze truncated AtNLP7-GFP proteins nuclear retention triggered by nitrate and H_2_O_2_, seedlings growing on medium with 10 mM KNO_3_ for 5 days were transferred to N-free medium for two days then treated with 10 mM KNO_3_ or 10 mM KNO_3_ + 0.5 mM H_2_O_2_ for 1 h. Laser scanning confocal imaging used Zeiss 880 microscope with argon laser (488 nm for green fluorescent protein (GFP) excitation).

### RNA extraction and qRT-PCR

RNA extraction, reverse transcription, and qRT-PCR were performed as described previously [5, 7]. Briefly, reverse transcription was performed using total RNA extracted with Trizol reagent (Invitrogen, Carlsbad, California, USA). qRT-PCR was conducted with StepOne Plus Real Time PCR System by using TaKaRa SYBR Premix Ex Taq II reagent kit. *UBQ5* was used as the internal control. The primers used are listed in Supplementary table. All materials used for RNA extraction were whole plants.

### Nitrate content and enzyme activity analysis

Whole 14-day-old seedlings grown on agar medium with 1 mM (LN) or 10 mM (HN) KNO_3_ were used for analysis. Nitrate was extracted in 50 mM HEPES–KOH (pH 7.4), and measured as described previously [37]. NR activity was analyzed with an enzyme-coupled spectrophotometer assay kit (SKBC, China) according to the manufacturer's guidelines.

### Uptake of ^15^ N-nitrate

^15^ N-uptake assay was carried out with ^15^ N-labeled KNO_3_ (99 atom % ^15^ N, Sigma-Aldrich, no. 335134). For ^15^ N-nitrate uptake experiment, 10-day-old seedlings grown in MS medium were pretreated with MS solution for 1 h and then transferred to 0.1 mM CaSO_4_ for 1 min. Then it was cultured in the modified MS solution with 5 mM K^15^NO_3_ as the only N source for 1 h, and finally returned to 0.1 mM CaSO_4_ for 1 min. The whole seedlings were dried to constant weight at 70 °C and ground. Used continuous flow isotope ratio mass spectrometer (DELTA V Advantage) and elemental analyzer (EA-HT, Thermo Fisher Scientific, Inc., Bremen, Germany) to analyze ^15^ N content.

### Chromatin immunoprecipitation

Seedlings were grown on MS medium for 10 days. The ChIP experiment was conducted as previously described [38]. The *nlp7-1* mutant, 35S:NLP7^∆NES^-GFP, 35S:NLP7^∆GAF−1^-GFP, 35S:NLP7^∆GAF−2^-GFP, 35S:NLP7^∆PB1^-GFP and pNLP7:NLP7–GFP transgenic plants, anti-GFP antibodies (Abmart), and salmon sperm DNA/protein A agarose beads (Millipore, USA) were used for ChIP assay. The DNA was purified and precipitated with phenol/chloroform (1:1, v/v). Used qRT-PCR to detect the degree of enrichment of DNA fragments. Enrichment values were calibrated with input DNA levels.

### Transient transactivation assay

Protoplast preparation and PEG transformation was performed as described before [39]. Different coding regions of *AtNLP7* derivatives were recombined into the pGreenII 62-SK vector as effectors, the entire promoter or the binding *cis*-element of target genes were recombined into pGreenII 0800-LUC vector as reporters. The dual luciferase reporter gene system was used for transient transactivation detection. Determined the relative activity of firefly luciferase (LUC) and renilla luciferase (REN), and computed the LUC/REN ratio. REN luciferase activity was used as an internal reference.

### NBT and DAB staining

The seedlings used for NBT and DAB staining were grown on 10 mM KNO_3_ for 5 days, transferred to nitrogen-free medium for 2 days and then treated with 10 mM KCl or KNO_3_ for 1 h. Plants were incubated with 1 mg/mL NBT solution or 1 mg/ml DAB solution at room temperature for 2 h and 6 h respectively, and then soaked in 80% (v/v) ethanol for 20 min before photographing to stop staining [28]. The signal intensity was quantified by Image J.

### Statistical analysis

Statistical analyses of the data were performed using analysis of variance (ANOVA). For multiple comparisons, one-way ANOVA with a Duncan post hoc test and Tukey’s honest significant difference test were used. For all statistical analyses, the difference was considered statistically significant when the *P* value < 0.05.

## Supplementary Information

**Additional file 1: Figure S1.** RT-qPCR analysis of *AtNLP7 *expression in WT, *nlp7-1* and the complementation lines. **Supplementary table**. Primers used in this study.

## Data Availability

All data and materials generated or analyzed during this study are included in this published article and its supplementary file. The sequence data used in this study can be found in the *Arabidopsis* Information Resource (https://www.arabidopsis.org/). The accession number of genes involved in the manuscript: *At4g24020* (*AtNLP7*), *At1g08090* (*AtNRT2.1*), *At1g77760* (*AtNIA1*), *At2g15620* (*AtNIR1*), *At5g35630* (*AtGS2*), *At5g67420* (*AtLBD37*), *At4g37540* (*AtLBD39*). The datasets and materials used during the current study are available from the corresponding author on reasonable request.
