# Supplementary material for: GAF domain is essential for nitrate-dependent AtNLP7 function
Source: BMC Plant Biol. 2022 Jul 25;22:366. doi: 10.1186/s12870-022-03755-x (PMC9310391; doi:10.1186/s12870-022-03755-x)
Supplement: Supplementary file 1 — Additional file 1: Figure S1. RT-qPCR analysis of AtNLP7 expression in WT, nlp7-1 and the complementation lines. Supplementary table. Primers used in this study. [file 12870_2022_3755_MOESM1_ESM.docx]

**Supporting information for GAF domain is essential for nitrate-dependent AtNLP7 function by Wu et al.**

**Figure S1. RT-qPCR analysis of *AtNLP7* expression in WT, *nlp7-1* and the complementation lines.** Seedlings were grown on MS medium for 10 days before RNA isolation from the whole seedling. Transcript levels were normalized against *AtUBQ5* expression. RT-qPCR data are mean ± SD (n = 3). P values are from the one-way ANOVA (The letters indicate significant differences. P < 0.05).

**Supplementary table: Primers used in this study.**

**Primer name** **Sequence （5’ to 3’）** **Note**

pNLP7-GFP-F CGGGATCCATGTGCGAGCCCGATGATAAT

pNLP7-GFP-R CTAGCTAGCTCACAATTCTCCAGTGCTCTC

pNLP7^∆NES^ -GFP-F CGGGATCCATGTCATGGCCACTAGATCAAATCC

pNLP7^∆NES^ -GFP-R CTAGCTAGCTCACAATTCTCCAGTGCTCTC

pNLP7^∆^^GAF-1^-GFP-F CGGGATCCATGGTAAATCTGAAAAGCTCGGAAAT

pNLP7^∆GAF-1^-GFP-R  CTAGCTAGCTCACAATTCTCCAGTGCTCTC

pNLP7^∆GAF-2^-GFP-F CGGGATCCATGGGGAAGACTGAGAAAACAATCA

pNLP7^∆GAF-2^-GFP-R CTAGCTAGCTCACAATTCTCCAGTGCTCTC

pNLP7^∆PB1^-GFP-F CGGGATCCATGTGCGAGCCCGATGATAAT **Vector**

pNLP7^∆PB1^-GFP-R CTAGCTAGCTCACGTTCTCATTTCTGAGCCTGAT **construction**

pGreenⅡ62sk/NLP7-F  CGGGATCCATGTGCGAGCCCGATGATAAT

pGreenⅡ62sk/NLP7-R ACGCGTCGACTCACAATTCTCCAGTGCTCTC

pGreenⅡ62sk/NLP7^∆NES^-F CGGGATCCATGTCATGGCCACTAGATCAAATCC

pGreenⅡ62sk/NLP7^∆NES^-R ACGCGTCGACTCACAATTCTCCAGTGCTCTC

pGreenⅡ62sk/NLP7^∆ GAF-1^-F CGGGATCCATGGTAAATCTGAAAAGCTCGGAAAT

pGreenⅡ62sk/NLP7^∆ GAF-1^-R  ACGCGTCGACTCACAATTCTCCAGTGCTCTC

pGreenⅡ62sk/NLP7^∆PB1^-F CGGGATCCATGTGCGAGCCCGATGATAAT

pGreenⅡ62sk/NLP7^∆PB1^-R ACGCGTCGACTCACGTTCTCATTTCTGAGCCTGAT

pGreenⅡ0800/NIR1pro-F ACGCGTCGACTCAATGCGGAAACTTGGATGTTATC

pGreenⅡ0800/NIR1pro-R CGGGATCCGATGATGGCGGAAGAAGGAG

AtNIR1-ChIP-NRE-F TGAAGTCAATAGACTAAGTGAATCA

AtNIR1-ChIP-NRE-R GATGATGGCGGAAGAAGGAGTT

AtNIR1-ChIP-Control-F TATGAAATCTATCTCCCTACATGC

AtNIR1-ChIP-Control-R CAAATGTTATGATATGGTAGGATTG **ChIP-qPCR**

AtNIA1-ChIP-NRE-F GTGATGCGTCCACTTGTTCATC

AtNIA1-ChIP-NRE-R TGGAGTTAAGCTATATGTTGCCG

AtNIA1-ChIP-Control-F GGGCAATATCGTGGGAGGGTA

AtNIA1-ChIP-Control-R GCCACAAACAATTCCGACACCA

AtNRT2.1-qRT-PCR-F CTCAATCCCCACCTCAGCTA

AtNRT2.1-qRT-PCR-R AACAAGGGCTAACGTGGATG

AtNIA1-qRT-PCR-F TTCCACTCCATTACGTCCGCAA

AtNIA1-qRT-PCR-R CGGGAAGATCCTCCGCTCCT

AtNIR1-qRT-PCR-F CCGGTAGCCAGTTCTGCG

AtNIR1-qRT-PCR-R CCTATTCGTCCCCCGACGT **RT-qPCR**

AtGS2-qRT-PCR-F CACCAAACCTTACTCTCTGACA

AtGS2-qRT-PCR-R CACTATCTTCACCAGGTGCTTG

AtLBD37-qRT-PCR-F TGCTTTGTTTCAGTCGTGCT

AtLBD37-qRT-PCR-R TGCTCCGTTAACTGGATTGACA

AtLBD39-qRT-PCR-F GAACTCCAACGTCCTGCTTTGT

AtLBD39-qRT-PCR-R ATACCAACCGCTCCGTTAACC

AtUBQ5-qRT-PCR-F AGAAGATCAAGCACAAGCAT

AtUBQ5-qRT-PCR-R CAGATCAAGCTTCAACTCCT
